# Towards a seamful ethics of Covid-19 contact tracing apps?

**DOI:** 10.1007/s10676-020-09559-7

**Published:** 2020-09-28

**Authors:** Andrew S. Hoffman, Bart Jacobs, Bernard van Gastel, Hanna Schraffenberger, Tamar Sharon, Berber Pas

**Affiliations:** 1 Interdisciplinary Hub for Security, Privacy and Data Governance (iHub), Radboud University, Nijmegen, the Netherlands; 2 Department of Practical Philosophy, Radboud University, Nijmegen, the Netherlands; 3 Institute for Computing and Information Sciences, Radboud University, Nijmegen, the Netherlands; 4 Open University, Heerlen, the Netherlands; 5 Institute for Management Research, School of Management, Radboud University, Nijmegen, the Netherlands

**Keywords:** Digital ethics, Seamful infrastructure, Critical design, Contact tracing, Covid-19

## Abstract

In the early months of 2020, the deadly Covid-19 disease spread rapidly around the world. In response, national and regional governments implemented a range of emergency lockdown measures, curtailing citizens’ movements and greatly limiting economic activity. More recently, as restrictions begin to be loosened or lifted entirely, the use of so-called contact tracing apps has figured prominently in many jurisdictions’ plans to reopen society. Critics have questioned the utility of such technologies on a number of fronts, both practical and ethical. However, little has been said about the ways in which the normative design choices of app developers, and the products that result therefrom, might contribute to ethical reflection and wider political debate. Drawing from scholarship in critical design and human–computer interaction, this paper examines the development of a QR code-based tracking app called Zwaai (‘Wave’ in Dutch), where its designers explicitly positioned the app as an alternative to the predominant Bluetooth and GPS-based approaches. Through analyzing these designers’ choices, this paper argues that QR code infrastructures can work to surface a set of *ethical–political seams*, two of which are discussed here—*responsibilization* and *networked (im)permanence*—that more ‘seamless’ protocols like Bluetooth actively aim to bypass, and which may go otherwise unnoticed by existing ethical frameworks.

## Introduction

In the early months of 2020, the deadly Covid-19 disease spread rapidly around the world. In response, national and regional governments implemented a range of emergency lockdown measures, curtailing citizens’ movements and greatly limiting economic activity. More recently, as jurisdictions across five continents have begun lifting these restrictions, the use of so-called contact tracing apps has figured prominently in plans to reopen society, alongside the more basic prerequisite of having capacity to conduct population-based testing for the disease at scale (WHO 2020).

The World Health Organization defines contact tracing as ‘the process of identifying, assessing, and managing people who have been exposed to a disease to prevent onward transmission’ and deems it ‘an essential public health tool for controlling’ Covid-19 and for ‘break[ing] the chains of transmission’ (ibid.). With contact tracing apps, this process can be partially or totally automated: it has been envisioned that users will voluntarily download and install software packages on their mobile phones which digitally track their interactions and/or movements. Once someone receives a confirmed diagnosis of Covid-19, that information can be transmitted via a remote infrastructure that would then determine what other citizens had been in close physical proximity of the diagnosed case while they were capable of shedding the disease and issue a warning to those other citizens’ mobile devices with further information and instructions, e.g. that they should get tested, monitor for symptoms, or enter self-isolation for the standard 14 day quarantine period. The general idea is that apps on phones locally collect information about contacts with other people. Only when a particular person is found to be infected does their phone release a ‘contact code’ to health authorities or another responsible party for the purposes of transmitting that to the server, and can then be used to warn others about possible exposure and aid in tracking down the initial source of the infection to stem further spread. A recent investigation tallied no less than 80 such apps that were either under development or being rolled out (Tokmetzis and Meaker 2020), with several different proposed solutions for how such contact-tracing functionality should be implemented, including those that rely on Bluetooth, GPS location data, and/or the use of barcode-like Quick Response codes (henceforth QR codes).

Regardless of the specific technological infrastructure that Covid-19 tracing apps deploy, a survey of the discourse within recent academic scholarship and media coverage about these apps reveals a consistent set of ethical concerns and critiques that detractors raise, falling along two primary axes: first, questions about the precise goals, efficiency, and usefulness of such apps towards their stated aim; and second, possible threats to individuals’ privacy and autonomy (e.g. Greenberg 2020; Lomas 2020). However, it is our contention that the discussions about ethical limitations of Covid-19 tracing apps as a class corresponding to these two axes threaten to elide a broader consideration of the values that are *embedded into the design of particular implementations of contact tracing apps* as well as those *normative actions these specific design decisions might provoke* among app users. It is these latter two concerns which are the focus of the present paper, and which we discuss through an examination of the development of a QR code-based contact tracing app called Zwaai. As we will show, Zwaai serves as an instructive site for unpacking the normativities of app design in such a way that moves beyond more generalized concerns about *threats* to individual privacy and autonomy, enabling a more granular view of the *values that both go into and, in turn, might emerge out of users’ engagement with digital technologies* like Covid-19 contact tracing apps.

To do so, we draw on the work of Inman and Ribes (2019) in order to situate Zwaai—and its use of QR code technology—as a ‘seamful’ approach to contact tracing app design, which stands in contrast to the more ‘seamless’ approaches suggested by the use of passive tracking technologies like Bluetooth and GPS data. The notion of ‘seams,’ which Andersson (2007) defines as ‘uncertaint[ies] in sensing and ambiguit[ies] of representations,’ is especially useful for thinking through the ethics of digital technologies like contact tracing apps because as a conceptual lens it surfaces the sets of values and concerns that are otherwise obscured amidst the normative work of designing mobile phone apps, and digital infrastructures more generally. In thinking through the relationship between *seams* and *values*, it is worth noting here that seamful design is both something that can be valued in its own right, insofar as it presents certain affordances beyond what more *seamless* alternatives may offer, but also that seamful design can serve as a vehicle for enacting certain ethical–political values (e.g. Friedman and Hendry 2019). In our analysis of Zwaai, we thus argue that its designers mobilize seamful design as a means of surfacing a set of what we refer to here as *ethical–political seams*, for which we have space to address only two: *responsibilization* and *networked (im)permanence*. For heuristic purposes, we can think of these two ethical–political seams as more or less coextensive with certain ‘values’ being worked upon by the architects of this particular contact tracing app. This means that we approach seamfulness only from the point of view of the designers, which leaves questions of user engagement and appropriation open for further investigation, a point to which we return in the conclusion.

The remainder of the paper proceeds as follows: in Sect. 2, we discuss the many justifications for and critiques of the use of GPS and Bluetooth technologies in contact tracing apps, drawing largely from popular press writings on the matter over the course of the first several months of 2020. Section 3 moves on to describe other approaches to contact tracing apps, namely the use of QR codes which have been proposed as an alternative infrastructure for facilitating contact tracing, and that we situate as an instance of seamful design. From there, we delve deeper into unpacking the two aforementioned *ethical–political seams* as a means of drawing out the ways in which seamful technologies like QR codes work to socio-materially enact particular normativities and ethical stakes. We then conclude with a discussion about the affordances of the twin concepts of *seamlessness* and *seamfulness* for conducting ethical analyses of Covid-19 contact tracing apps in particular, and mobile health technologies more broadly. In sum, our overall aim is to move past the dominant proccupations with utility, privacy, and autonomy found within existing ethical frameworks and to instead shift the focus to how seamful design—in this case, enacted in the QR code-based infrastructure of Zwaai—is mobilized in developing contact tracing apps, and to the design work that is undertaken in the process of surfacing specific *ethical–political seams*.

Before moving on to flesh these ideas out in greater depth, and in lieu of a methods section –note that Zwaai has been developed as a prototype app by members of the Interdisciplinary Hub for Security, Privacy and Data Governance (iHub) at Radboud University Nijmegen in the Netherlands.^1^ The context for its development was a call for submissions, published by the Dutch government’s Ministry of Health, Welfare and Sport in April 2020, which sought proposals for contact tracing apps that could be used as part of the reopening strategy in the Netherlands. Only seven of the more than some 700 submissions were selected to move on to a weekend-long ‘appathon’, held in late April, where experts were to evaluate the prototypes and would then ultimately move on to further developing the best candidate; Zwaai was not one of them. Despite the surfeit of reflexive ethical considerations expressed throughout the event (Verbeek 2020), the appathon was widely criticized for its hastiness and lack of transparency of evaluative criteria (Wassens 2020). In the end, the Ministry of Health ultimately shelved the idea of using a third-party app and has since undertaken its own in-house development of a contact tracing app called Coronamelder.

## Justifying and critiquing seamless design: Bluetooth and GPS protocols

One of the most striking features of Covid-19 contact tracing apps during the first few months of 2020 has been the emphasis placed on the use of GPS and Bluetooth technologies as the go-to infrastructures for automating the work of contact tracing. National and state governments in China, Iceland, India, Korea, and the United States were counted among the first movers in the development of contact-tracing software, all of which sought to use automated GPS tracking that leverages geolocation data of mobile phones to monitor the spread of Covid-19 and to identify ‘hot spots’ of the virus (Servick 2020). Meanwhile, Australia’s COVIDSafe app—first launched in late April 2020—aimed to collect ‘anonymous IDs’ from the smartphones of app users who have been within transmitting distance of each other for at least 15 min, requiring that users keep their Bluetooth signal activated at all times (Kang and Haskell-Dowlad 2020). Also in April 2020, the United Kingdom’s National Health Service launched its own pilot Bluetooth tracing app with an overall similar set of functionalities as the former: app users exchange ‘anonymous’ keycodes via Bluetooth signal and if an individual tests positive, they use the app to ping a centralized database with confirmation of diagnosis, whereupon the individual user’s ID is matched with all other IDs who had been in transmitting distance of the case, and notification is then sent to ‘others judged to be at high risk of contagion’ (Kelion 2020a, b). Apps using Bluetooth-based protocols have also been developed for use in countries as diverse as Singapore, France, Hungary, and Malaysia, while several others, including Qatar, Turkey, and Norway, have apps that combine GPS and Bluetooth protocols (O’Neill et al. 2020).

Justifications for the use of GPS and/or Bluetooth technologies in contact tracing apps are largely couched in a vernacular of technological efficiency (e.g. Boltanski and Thévenot 2006), which is said to work in two directions. On the one hand, it can aid in the work of public health experts who, as previously noted, typically must carry out contact tracing in a very manual sense—calling infected persons, gathering information about who they may have come in contact with, then reaching out to those who have possibly been exposed and rendering advice on next steps to take. Delegating some of this work to digital infrastructure reduces some of the workload for human contact tracers, allowing them to refocus on certain tasks. On the other hand, some argue that there are efficiencies for citizens themselves, and this is especially true of contact tracing apps that rely on more ‘passive’ mechanisms like Bluetooth and GPS, which simply require the app user to install the app on their mobile phone device and to activate its Bluetooth or location settings. From here, the work of establishing proximity to other app users is handled in a mostly automatic fashion, with the main point of human intervention coming only when a user needs to report a positive diagnosis to the system, which can in theory be done either directly by the end-user, or by an approved public health expert.

Terms such as ‘digital handshakes’ or ‘Bluetooth handshakes’ point to the ability for mobile devices to automatically register contacts without the need for any immediate human intervention (Bacchi 2020). In rendering his own technological imaginary of such a scenario, Bill Gates described a tool that ‘would involve using Bluetooth plus sending a sound out that humans can’t hear but that verifies that the two phones are reasonably close to each other’ (Gates 2020). This work of de-emphasizing human interaction with Covid-19 contact tracing apps can be thought of in terms of what Inman and Ribes (2019) describe as ‘seamless design,’ an approach to the design of computational artefacts that ‘emphasizes clarity, simplicity, ease of use, and consistency to facilitate technological interaction’ (p. 2) and whose advocates have historically ‘cast [it] as a virtue through a conceptual coupling with lowered cognitive load, and thus freedom’ (p. 7). As we have hinted at in the introduction, the issue of freedom is in fact paramount to understanding why contact tracing apps have gained so much traction in the first place: proponents argue that the ability to do contact tracing at scale is perhaps the foremost antidote to lifting government mandated lockdowns which greatly restricted individuals’ movements and, in the eyes of some critics, constitute human rights violations (Delvac 2020). And so, in the emergence of contact tracing apps, we find a co-articulation of a freedom from cognitive load and a freedom of movement.

But as quickly as countries began announcing the development of Covid-19 contact tracing apps as a path (back) to the freedom of movement, detractors began publicly contesting their roll-out, with critiques largely focusing on three core issues. From a practical standpoint, critics raised questions about the overall accuracy and precision of both Bluetooth and GPS, which can contribute to both false positives and false negatives, as well as questions of performance. For instance, the requirement for apps to be actively opened on users’ phones is known to drain phone batteries and to interfere with other apps running simultaneously (Tokmetzis and Meaker 2020). Others have questioned the overall effectiveness of apps in preventing the spread of Covid-19 in the first place, including some who have actively advocated for their use. Singapore’s TraceTogether app has been flagged as a case where early uptake by residents in early March 2020—totaling over 600,000 downloads in the first three days after the app was released—nevertheless failed to prevent a spike in infections later that same month (Lee 2020). Officials overseeing contact tracing efforts in Iceland—which has been said to have ‘the highest penetration of any automated contact tracing app in the world’ with 38% of citizens using its Rakning C-19 app—have meanwhile claimed that the app ‘wasn’t a game changer for us’ (Johnson 2020).

In terms of addressing the ethics and ethical limitations of using mobile phone apps for contact tracing, issues around their privacy and security have been another major avenue for criticism. On the user-facing side, those who have looked at apps across a number of jurisdictions have found that many of them do not spell out their privacy and data management policies at all, while others contain quite vague policy language (Tokmetzis and Meaker 2000). Looking more closely at the digital infrastructures supporting contact tracing, an additional set of critiques have been raised regarding governments’ use of centralized servers or databases to store information. Proponents argue that centralized strategies have the advantages of being able to check in to ensure the right individuals are being notified of a possible exposure; to follow up on whether users who received an alert on their phone ultimately develop symptoms and/or test positive for Covid-19; and to modify the technical functioning of the apps based on analyses of faulty Bluetooth ‘handshakes’ that did not result in a notification where one should have been issued (Servick 2020). However, this comes with giving governments access to highly sensitive personal information about app users, including their movements, who they have come into contact with, and their diagnostic status. In cases where such centralization is in play, this is required for then transmitting messages about possible contagion and what next steps those contacts should take (e.g. entering self-isolation or quarantine for a given period of time.)

Such centralization opens the door for any number of ‘bad actors,’ including hackers and private sector actors who gain access to contact tracing app data, to use this information to reconstruct a user’s ‘social graph,’ including details about who a user came into contact with, as well as details about where and when those interactions occurred. Compounding this is the possibility of ‘mission creep’—in other words, the likelihood that not bad actors, but governments themselves, could extend the use Bluetooth and GPS-based contact tracing, whose use is initially justified during the current pandemic, to other more nefarious surveillance purposes even once the pandemic has subsided (Busvine 2020). Consider, for instance, the publication of a Joint Statement on Contact Tracing in April 2020, signed by over 300 researchers (largely representing the health and computer sciences) from 25 countries. In the statement, its signatories warn that some Bluetooth-based proposals threaten to ‘enable (via mission creep) a form of government or private sector surveillance that would catastrophically hamper trust in and acceptance of such an application by society at large,’ going on to argue that ‘[i]t is vital that, in coming out of the current crisis, we do not create a tool that enables large scale data collection on the population, either now or at a later time’ (‘Joint Statement’ 2020).

It should be noted here that there is nothing inherent to Bluetooth-based contact tracing apps that requires such a centralized approach, however. Whence the ‘unprecedented collaboration’ announced in April 2020 between Google and Apple, where both firms together developed a cross-platform API—the Google Apple exposure notification (GAEN)—that effectively exposes the operating systems of their respective Android and iPhone mobile phone operating systems for the purposes of enabling a more *decentralized* approach to contact-tracing app development atop those platforms (Hern and Paul 2020). For many, this was a welcome contribution to the contact tracing app ecosystem and by late May 2020, over 20 countries across 5 continents had requested the API (Fisher 2020). The United Kingdom, which had initially rejected using the GAEN one month prior to its release, relented in early June and announced that it would in fact be switching to that protocol (Kelion 2020a, b), while the Dutch Ministry of Health, Welfare and Sport has also developed its own in-house app atop the GAEN as well (Wokke 2020).

Despite the improvements to privacy and security that this solution offers against many of the previous home-grown attempts to develop apps, an additional set of ethical questions has been raised which aim to go beyond considerations of privacy and security alone. One major concern here is that seamless technologies may lead to something of an autonomy vacuum, where citizens may delegate the work of preventing spread of Covid-19 to the apps, and may thus be more inclined to participate in risky behaviors—like gathering in large groups, or in enclosed spaces—with the assumption that the app will simply notify them later if they have been in contact with a confirmed case (Anonymous 2020). Additionally, several commentaries have also raised questions about the possible effects of handing off control of digital infrastructure to private, for profit firms like Google and Apple. This includes critiques about the closed nature of API development among these parties (Meaker and Tokmetzis 2020), which goes against the central tenets of the open software movement, and larger political economic questions about the outsourcing of digital infrastructure to for-profit entities and the accompanying shifts in societal values that might accompany such a move (Sharon 2020).

## Beyond Bluetooth and centralization: QR codes as seamful design?

In an effort to circumvent many of the aforementioned concerns addressed in the previous section, app developers have also proposed technological designs which rely on neither Bluetooth nor GPS technology, with Quick Response (or ‘QR’) code-based infrastructure being one leading alternative in this space. QR codes are a type of machine-readable, two-dimensional matrix barcode that can be encoded with information. Most current smartphones are equipped with QR code reading capabilities, and so when a phone user scans a QR code – say, that is printed on the back of a product box or that appears on a billboard pasted in a public transportation system—the phone will perform some kind of operation upon scanning, such as redirecting the phone’s internet browser to a company website where the user can get further information about the advertised product. In the context of the present paper, the use of QR codes for contact tracing doesn’t launch a browser but rather triggers an app installed on a user’s phone to record certain information encoded in the QR code itself, such as the location where the code was posted (at a specific grocery store, for example) and the exact time when the code was scanned.

Singapore’s ‘SafeEntry’ app was one of the earliest instances of countries deploying QR code-based infrastructure to support Covid-19 contact tracing activities, amidst wider efforts to reopen society following the first wave of infections (Sharwood 2020). First launched in mid-May 2020, the Singaporean government mandated all businesses and public venues, including parks, malls, and train stations to post QR codes at their entrances and citizens were required to scan the code upon entering and again upon exiting these locations. Information about the location and time a QR code was scanned, along with users’ names, phone numbers, and national identity numbers, are then sent remotely to a centralized cloud-based storage system operated by the Singaporean government, which they could then use for the purposes of contact tracing; this is an instance of a centralized strategy of data storage. Other more decentralized and/or privacy-preserving QR code infrastructures have also been proposed, including a prototype peer-to-peer contact tracing app called TrackCOVID, which uses a ‘transmission graph’ data structure for tracking possible exposures to Covid-19 within interaction networks (Yasaka et al. 2020) as well as the Zwaai app we describe in greater depth below. Importantly, regardless of whether QR codes invoke a centralized or decentralized data storage paradigm, a key commonality across all instances of QR infrastructures is that their utility and effectiveness ultimately depend upon the active agency of users, who must scan a QR code in order to initiate some activity on their smartphone.

As of late May 2020, of the approximately 80 contact tracing apps that Dimitri Tokmetzis and Morgan Meaker have tallied up as part of their worldwide investigation into Covid-19 tracing apps for the digital magazine *The Correspondent*, some 15 of those are reported to rely on QR codes alone (and another 4 combing QR codes in tandem with Bluetooth and/or GPS.) Drawing on examples of the use of QR-based contact tracing by governments in Malaysia, New Zealand, Singapore, and Thailand, the authors discuss the affordances of such technology as compared with its Bluetooth or GPS-based counterparts, which include the relative technological simplicity of QR codes—which makes implementation easier and speedier—and also the fact that QR-based systems fit better into existing work routines in settings where contact tracing is still a manual, human-led endeavor, as is the case in many other jurisdictions). The downsides of QR-based apps are that they are only as effective as humans’ compliance; namely, their willingness to scan a code when they enter and exit a building (like a grocery store or a shopping mall); these provide only a fairly low resolution of proximity, e.g. knowing an infected person was in a large space like a shopping mall cannot tell you if others walking around the mall actually came into contact with that individual; and that, unless data transmission is totally encrypted and decentralized, so too might QR code apps be open to the same privacy and security breaches as Bluetooth and GPS. In other words, it is possible to have a centralized QR system that links app users’ unique identifiers with other information like national ID numbers and phone numbers.

It is of course important to acknowledge the affordances of approaches that go beyond the technical and ethical limitations of Bluetooth and GPS discussed in the previous section. However, we contend that by emphasizing the efficiencies for implementation and the possible behavioral and informational shortcomings that QR code-based systems present, QR infrastructures are measured against an implicit standard of seamlessness, which has historically devalued more seamful approaches (Inman and Ribes 2019). In an effort to move past evaluations which are biased toward seamlessness, our aim in this section is thus to resituate QR codes as an instance of ‘seamful design,’ which we contrast with the more ‘seamless’ Bluetooth and GPS-based systems discussed in the previous section. In doing so, our objective is to highlight the role that seamful design can play in surfacing specific *ethical–political seams* that more ‘seamless’ protocols like Bluetooth actively aim to bypass, and which may go otherwise unnoticed by existing ethical frameworks. To do this, we investigate the design of the aforementioned prototype contact tracing app called Zwaai—the Dutch word for ‘Wave’—which was developed by several members of the Interdisciplinary Hub for Security, Privacy and Data Governance (iHub) at Radboud University Nijmegen in the Netherlands.

As we have just said, Zwaai relies on a QR code-based infrastructure to do contact tracing. Each user’s app produces a continuously refreshed QR code and also the ability to scan other users’ QR codes; it is the QR code that helps keep track of what interactions a given app user has had, albeit in a privacy-preserving way as there is no unique identifier assigned to any individual user or their app. The app developers have in fact presumed that there are two broad classes of interactions we have in the world. In the first, which they call a ‘wave’—hence the name of the app—a user might meet with a friend or a colleague—say, for dinner at someone’s house or for a drink in the park. In such a setting, the different parties scan each others’ QR codes when they meet, as they would shake hands upon greeting each other (or even give the customary three cheek-kisses in line with Dutch tradition). In doing so, the phones exchange random numbers which are locally stored (in other words, decentralized) on the user’s device for a given period of time along with information about the time and duration of the interaction. A similar process takes place in the second class of interactions, which is when one enters a building—for instance, a supermarket, a train car, or one’s office. Here, rather than scan another person’s QR code, though, the building itself is equipped with its own unique QR code that app users are expected to scan upon entering, and again upon leaving, the building. Organizations can obtain a QR code which they are then expected to register with the entity responsible for administering the system.

In effect, each app user can be thought of as being subscribed to a central server. If an individual receives a positive diagnosis for Covid-19, a responsible party, such as a public health physician, can then publish this information to the server with the approval of the diagnosed user. Individual users’ apps routinely ping the server looking for matches between the set of locally stored random numbers, and those that correspond to a positive diagnosis. If a match is found, regardless of whether it was via a ‘wave’ or if a user has visited a ‘contaminated’ building such as a supermarket, the system will automatically notify the user that they may have been in contact with a confirmed case of Covid-19 and can provide additional advice, such as to get tested or to undergo 14-day self quarantine. This decentralized infrastructure has specifically been designed in such a way as to ensure users’ privacy, as the entire system is based on locally stored random numbers that cannot be traced back to individual users. It also gives public health authorities insight into possible ‘heat zones’ where new clusters of infections may be taking place.

As is clear from this brief description, Zwaai is not altogether dissimilar from how many public transport systems require you to ‘wave’ your transit card over a reader, which registers when you enter (and sometimes when you exit) the system. As it has been envisioned, users of Zwaai would be expected to use their phone’s QR code reading capabilities and to ‘wave’ it over a QR code—posted at the entrance to a building, or that is generated by another user’s Zwaai app—to register the location or other app user(s) with whom they have been in close physical contact. As such, the app actually requires users to physically maneuver their phone over the code in order to register the contact. It is for this reason that we qualify Zwaai as a case of *seamful design*. Whereas seamless design values things like simplicity and ease of use, seamful design ‘emphasizes configurability, user appropriation, and *revelation of complexity, ambiguity, or inconsistency*’ (Inman and Ribes 2019, p. 2). Thus, in contrast to the seamlessness discussed in the previous section, Zwaai instead asks that users actively engage with the contact tracing infrastructure in a much more embodied way than what Bluetooth or GPS otherwise demands. But the question can then be raised as to the designerly intentions of those who have developed the Zwaai app: what can we learn or say about the ethics of Covid-19 contact tracing apps more generally from looking at this one specific case?

## Unpacking the *ethical–political seams* of the Zwaai app

Our response to the question just raised is that the language of *seams* allows us to more precisely respecify our analysis in terms of the norms and ethical positions that inform technology design as well as the particular types of behaviors and debates that such technologies might provoke among users. Looking more closely at how the developers of Zwaai (included among the authors of this paper) have discussed the app—with the below-quoted excerpts taken from an unpublished proposal submitted for consideration in the aforementioned appathon, as well as from journalistic coverage about the app—we focus here on only two *ethical–political seams* which it aims to surface: *responsibilization* and *networked (im)permanence.* These are of course by no means the only seams that Zwaai might bring to the fore, and in the concluding section of the paper we return to a discussion about what other seams such an app might make visible, and a corresponding set of values it might otherwise enact.

### Responsibilization

The first such seam that Zwaai’s designers have aimed to enact is what we shall call *responsibilization*. In their discussion of seamlessness, Inman and Ribes point out that to be seamless is not necessarily to be invisible, but rather ‘to be compatible, mundane, interoperable’ (2019, p. 9). It is precisely such outcomes that contact tracing technologies using Bluetooth or GPS aim to achieve, as phones conduct ‘digital handshakes’ with one another and with a server in the cloud beyond the immediate perception of users. Yet as the developers of Zwaai explain it: ‘With contact apps based on Bluetooth, it is only an on/off choice without further control over all links that are *invisible.*’ This is consequential on a number of fronts.

For one, the discourse around controlling Covid-19 in the Netherlands—and promoted by the Dutch Prime Minister, Mark Rutte, in his many public appearances relating to the Covid-19 pandemic—stresses the central role of ‘autonomous individuals’ taking responsibility and being accountable for their own actions, which Rutte has claimed is a hallmark of a ‘mature democracy.’ For the developers of Zwaai, then, the seamlessness of Bluetooth and GPS contravenes the predominating logics of Dutch governance, positioning citizens ‘as an unreliable herd that needs to be monitored,’ which, in turn, could dramatically (and negatively) impact citizens’ willingness to use an app if they sense that their agency and autonomy is being eclipsed. This is especially true of apps that use a centralized approach to data collection and storage, where citizens can be literally monitored at the individual level; but even with decentralized alternatives that have subsequently been developed—including the European DP3-T and aforementioned GAEN protocols—citizens may nevertheless operate on the assumption that they are being monitored, even if the technology does not allow for the identification of specific individuals through the types of data that are collected and exchanged.

A second worry is that the seamlessness of Bluetooth may also lead to a situation where app users delegate total responsibility to the technology itself, giving way to passive and careless behaviors, a reduced awareness of the likelihood of contagion, and a false sense of security (Farronato et al. 2020; van Dongen 2020). This so called ‘risk-compensation hypothesis’ suggests that policies and regulation and/or technology designed to protect from harm (such as the requirement that automobiles be equipped with safety belts and laws requiring their use) has a contrary effect on safety, as people believe to be protected and thus behave more recklessly (Houston and Richardson 2007).^2^ Conversely, the developers of Zwaai claim that the QR code infrastructure that their app mobilizes is in fact much more in line with Covid-19 policy measures in the Netherlands and its emphasis on transparency and the role of individual citizens.

Here, they stress the *seamfulness* of its architecture: by using Zwaai, citizens are ‘responsible for their warning network,’ which ‘requires an active attitude and contribution.’ Users are—at least to a certain extent—positioned to have ‘control’ and ‘direction’ over ‘who [or] what they include in their network.’ In other words, the very act of scanning a QR code when meeting with a friend, or when entering the supermarket, is not just a visible but also a socio-materially enacted reminder that one is living amidst an ongoing health crisis and that the risk of contagion is still very real—even as rates of transmission may be waning. Interfacing with this infrastructure also enacts the user’s agency and accountability, and by extension, their responsibility for participating in limiting the spread of Covid-19. That said, the full dependence upon user agency within the context of Zwaai may also be perceived as a limitation, as we will address shortly—namely, that the imperative to constantly engage with the infrastructure may lead users into a state of fatigue or even annoyance, as well as the fact that the system does not scale up very easily. While Zwaai’s designers may view these as *features* of the app and its extant infrastructure, they are equally likely to be experienced as *bugs*, ultimately impacting the likelihood that Zwaai or similar QR code-based contact tracing infrastructures get taken up in the first place.

### Networked (im)permanence

Positioning users as active participants in the contact tracing infrastructure, as just discussed, contributes to the enactment of a second ethical–political seam of *networked (im)permanence*. As the developers discuss in their proposal, the seamlessness of Bluetooth and its remote signaling capabilities opens such an infrastructure up to interception by third parties—what have been described above as ‘bad actors’—who can repurpose and rebroadcast that data, for instance in other locations where a given individual has not been physically present. Such malicious activities can contravene contact tracing efforts and even contribute to the delegitimation of contact tracing apps in the eyes of the public.

According to its designers, Zwaai has been intentionally designed for the purpose of ‘issuing warnings about the true proximity to contaminations within self-created networks.’ The idea of ‘true proximity’ here speaks to the seamfulness of the QR code system that Zwaai deploys and the fact that Zwaai connections ‘must be made by users themselves.’ Thus, while Bluetooth or GPS may offer a higher resolution view of app users’ physical proximity to one another, there are still significant performance issues with these technologies, leaving open the possibility for registering false positives or false negatives. Zwaai, on the other hand, in activating users’ agency in scanning a QR code, provides a definitive statement about a given contact—if a user scans a QR code, it gets logged by the app, leaving much less room for false positive or false negatives. In this way, Zwaai can be likened almost more to a semi-automated diary that keeps track of where a user has been (when they scan a code at the entrance to a business) or with whom they have interacted (when they scan another users’ QR code when meeting for a coffee). An additional benefit of this infrastructure is that it also significantly limits the likelihood that the transmitting of a Bluetooth signal can be maliciously repurposed or rebroadcast for other ends (Cyphers and Gebhart 2020).

Furthermore, the activation of users’ agency in this way also provokes an awareness among app users about the extent to which they are connected to the digital contact tracing network. Inman and Ribes’s historical overview has found that network connectivity has in fact been a central area of concern in human–computer interaction research into seamful design. According to these authors, seamfulness has been deployed as ‘a strategy for overcoming the black-boxing of seamlessness,’ one that focuses ‘on revealing disconnection in spatial relations’—a strategy motivated by designers’ desires to ‘[counter] the assumption that all users will desire constant connection to the network’ (p. 9).

Thus, in contrast to Bluetooth, the Zwaai infrastructure materially embeds this assumption about non-connectivity, delegating to the user—in the short term, while Covid-19 infection is still a very real possibility—the task of deciding exactly when and where to invoke the network rather than demanding constant connection. This also extends over a longer time horizon, as well. In a recent article about Zwaai, the author cites the viewpoint of one of the main Zwaai developers that ‘the annoyance that QR codes cause for some users is actually an advantage’ (Anonymous 2020). It goes on to quote the developer as saying, ‘I believe in human laziness: this system does not scale up, so it essentially self-destructs. You do not have the risk that the app will continue to collect user data in the background after the corona crisis, a risk that you do have with Bluetooth apps’ (ibid.). In other words, the imaginary at play here is that we may well one day again live in a world where the transmission of Covid-19 is no longer a reality. At that point, friends can decide to cease ‘waving’ with their apps when they meet each other, and organizations and grocery stores can pull down the QR codes that had been pasted at their entrances. In so doing, the infrastructure effectively ceases to exist. This is very different from other possible future scenarios where the operating systems of mobile phones have automatically been updated to include the capabilities for enacting Bluetooth-based tracking, which are much more likely to undergo a process of technological lock-in (David 1985) and thus are much less likely to be removed during subsequent operating system updates—leaving open the possibilities for future data breaches.

## Conclusion: towards a seamful ethics of Covid-19 contact tracing

As we have argued above, much of the existing scholarship and ethical reflection on the emergence, design, and deployment of Covid-19 contact tracing apps has tended to focus on a rather limited number of possible threats and overflows that such digital technologies present to society. In our view, this funneling of concern and critique risks overlooking the equally consequential work of designing mobile health technologies. Drawing on the discourses of *seamlessness* and *seamfulness* (Inman and Ribes 2019), our aim has been to provide a more granular account for the designerly normativities that developers embed in their contact tracing tools, and the socio-material means by which these apps reveal particular *ethical–political seams*.

Although we have only had space to address two of these seams—*responsibilization* and *networked (im)permanence*—it is clear that our work is not quite finished. Indeed, questions about the implications of individuals’ autonomy, and the privacy and security of their personal data, are themselves revelatory of a funneling of concern mentioned above. And yet as more and more research is showing, these issues do not exhaust the full spectrum of ethical–political-economic implications that new digital technologies engender and thus beg further, and more nuanced, analysis (e.g. Barocas and Levy 2019; Cohen et al. 2020; Sharon 2016). A central affordance of the heuristic of *seams* is its extensibility in accounting for what is revealed or otherwise hidden in the process of developing digital tools and infrastructures such as mobile phone applications. Another affordance stems from the seamful technology’s performativity, which positions users to become more aware of their actions.

In this regard, future research into the ethical–political dimensions of Covid-19 contact tracing apps would be well served by continuing to unpack their (in)visibilities and the designerly normativities which propel decisions about what seams reveal and what shall be relegated to the realm of seamlessness. In particular, questions about what *other* values and user behaviors might be at stake in the design and use of such tools—such as solidarity (Prainsack and Buyx 2017) or logics of care (Mol 2008) —are instructive to ask here. Additionally, attention to how users themselves—whether actual or potential—experience these technologies is key to refining our understandings about their ethical import, a task best left to the ethnographers and empirical ethicists among us (Pols 2015). Finally, there are many unanswered questions about other types of frictions that seamful design introduces—whether between users, technologies, and their material environments (Terpstra et al. 2019) or between agonistic and consensus-based political discourses (Disalvo 2012) —and what these frictions might hold: for users, for public health, and for further fostering ‘mature democracy’ in contemporary society.

## Data Availability

Not applicable.

## Footnotes

1 The analysis presented here is part of ongoing work crossing multiple research streams within the Interdisciplinary Hub for Security, Privacy and Data Governance (iHub) at Radboud University Nijmegen, in the Netherlands. As its name suggests, the iHub is a space that brings together a diversity of disciplinary expertises, and an interesting (and perhaps curious) feature of this paper is that the ideas have developed iteratively as the Zwaai concept has ‘traded hands,’ so to speak: among computer scientists, software developers, and user-centered design researchers who originally developed the prototype Zwaai app; philosophers of technology, who think deeply about the ethical stakes of digitalization, especially in the healthcare domain; and social scientists, including those working in science and technology studies and organization studies, who are attuned to the practical and epistemic implications of new digital tools. In a sense, then, the Zwaai app—and the many issues it raises—hits the ‘sweet spot’ of interdisciplinarity that the iHub has strived to hone since being launched in late 2018. One of the advantages of this colocation of diverse communities of practice at the iHub is that it provides an ideal environment for ‘reading’ work being done by one community through the lens of complementary perspectives culled from other communities—work that may be entirely relevant, but perhaps lying just beyond the field of legibility—leading to emergent collaborations amongst members of the center. It is precisely such an exercise that the present paper comprises, wherein the author has combined technical and conceptual work done by the app developers with ethical reflections proposed by our resident philosophers, and mediated these through insights culled from scholarship in infrastructure studies, (user-centered) design, and human–computer interaction. In doing so, we are led to re-articulate shared, but oftentimes unstated and even unrecognized, concerns in such a way that each of these communities are mutually enriched in this process of exchange.

2 Despite the fact that several studies have questioned or outright rejected the veracity of such a hypothesis (e.g. RIVM 2020), it has nevertheless been deployed at certain points in the public debate in the Netherlands about whether residents should be mandated to wear face masks in public spaces (Cohen 2020; Cornell 2020).

## References

[CR1] Andersson, K. (2007). Seamful Design in a Seamful Society. Paper presented at SIDER 2007, the 3rd Scandinavian Student Interaction Design Research Conference, March 8–9, Ronneby, Sweden.

[CR2] Anonymous. (2020, 12 May). Zwaai.app is an alternative for corona apps that require Bluetooth. *Radboud Recharge*. Retrieved June 2, 2020, from https://www.radboudrecharge.nl/en/article/zwaai-app-is-an-alternative-for-corona-apps-that-require-bluetooth.

[CR3] Bacchi, U. (2020, 5 May). Digital handshake: Can contact tracing deliver on its promise in coronavirus battle? Reuters. Retrieved June 23, 2020, from https://www.reuters.com/article/us-health-coronavirus-tech-trfn-idUSKBN22H254

[CR4] Barocas, S., & Levy, K. (2019). *Privacy Dependencies* (SSRN Scholarly Paper ID 3447384). Social Science Research Network. https://papers.ssrn.com/abstract=3447384

[CR5] Boltanski L, Thévenot L (2006). On justification: Economies of worth.

[CR6] Busvine, D. (2020, April 20). Rift opens over European coronavirus contact tracing apps. *Reuters*. Retrieved June 8, 2020, from https://www.reuters.com/article/us-health-coronavirus-europe-tech-idUSKBN2221U0

[CR7] Cohen, J. (2020, 12 July) The complex global evolution of coronavirus mask rules. *Forbes.*Retrieved August 12, 2020, from https://www.forbes.com/sites/joshuacohen/2020/07/12/the-complex-global-evolution-of--coronavirus-mask-rules/

[CR8] Cohen, J. E., Hartzog, W., & Moy, L. (2020, June 17). The dangers of tech-driven solutions to COVID-19. *Brookings*. Retrieved June 18, 2020 from https://www.brookings.edu/techstream/the-dangers-of-tech-driven-solutions-to-covid-19/.

[CR9] Cornell, M. (2020, 14 August)*.*The cure for the Netherlands’ false sense of security is masks. *The Holland Times*. Retrieved August 24, 2020, from https://www.hollandtimes.nl/articles/national/the-cure-for-the-netherlands-false-sense-of-security-is-masks/.

[CR10] Cyphers, B. & Gebhart, G. (2020, 28 April). Apple and Google’s COVID-19 Exposure Notification API: Questions and Answers. Retrieved August 16, 2020, from The Electronic Frontier Foundation. https://www.eff.org/deeplinks/2020/04/apple-and-googles-covid-19-exposure-notification-api-questions-and-answers.

[CR11] David PA (1985). Clio and the economics of QWERTY. The American Economic Review.

[CR12] Delvac, K. S. (2020, 25 May). Human Rights Abuses in the Enforcement of Coronavirus Security Measures. *The National Law Review.* Retrieved June 23, 2020, from https://www.natlawreview.com/ article/human-rights-abuses-enforcement-coronavirus-security-measures

[CR13] Disalvo C (2012). Adversarial design.

[CR15] Farronato, C., Iansiti, M., Bartosiak, M., Denicolai, S., Ferretti, L., & Fontana, R. (2020, 15 July). How to get people to actually use contact-tracing apps. *Harvard Business Review*. Retrieved August 22, 2020, from https://hbr.org/2020/07/how-to-get-people-to-actually-use-contact-tracing-apps

[CR16] Fisher, C. (2020, May 20). Apple and Google’s COVID-19 contact tracing tech is ready. *Engadget.* Retrieved June 22, 2020, from https://www.engadget.com/apple-google-covid-19-contact-tracing-api-170057362.html

[CR17] Friedman B, Hendry DG (2019). Value sensitive design: Shaping technology with moral imagination.

[CR18] Gates, B. (2020, 23 April). The first modern pandemic. *GatesNotes - The Blog of Bill Gates.* Retrieved June 22, 2020, from https://www.gatesnotes.com/Health/Pandemic-Innovation

[CR19] Greenberg, A. (2020, April 17). Is Apple and Google’s Covid-19 contact tracing a privacy risk? *Wired*. Retrieved June 18, 2020, from https://www.wired.com/story/apple-google-contact-tracing-strengths-weaknesses/

[CR20] Hern, A., & Paul, K. (2020, April 10). Apple and Google team up in bid to use smartphones to track coronavirus spread. *The Guardian*. Retrieved June 2, 2020, from https://www.theguardian.com/world/2020/apr/10/apple-google-coronavirus-us-app-privacy.

[CR21] Houston DJ, Richardson LE (2007). Risk compensation or risk reduction? Seatbelts, state laws, and traffic fatalities. Social Science Quarterly.

[CR22] Inman, S., & Ribes, D. (2019). “Beautiful Seams”: Strategic revelations and concealments. *Proceedings of the 2019 CHI Conference on Human Factors in Computing Systems*, 1–14. 10.1145/3290605.3300508

[CR23] Johnson, B. (2020, 11 May). Nearly 40% of Icelanders are using a covid app—and it hasn’t helped much. *MIT Technology Review*. Retrieved June 23, 2020, from https://www.technologyreview.com/2020/05/11/101541/iceland-rakning-c19-covid-contact-tracing/

[CR24] Joint Statement on Contact Tracing. (2020, 19 April). Retrieved June 8, 2020, from https://giuper.github.io/JointStatement.pdf.

[CR25] Kang, J. J., & Haskell-Dowland, P. (2020, 7 May). How safe is COVIDSafe? What you should know about the app’s issues, and Bluetooth-related risks. *The Conversation*. Retrieved June 2, 2020, from https://theconversation.com/how-safe-is-covidsafe-what-you-should-know-about-the-apps-issues-and-bluetooth-related-risks-137894.

[CR26] Kelion, L. (2020, 4 May). App stores approve UK contact-tracing app for test. *BBC News*. Retrieved June 2, 2020, from https://www.bbc.com/news/technology-52532435.

[CR27] Kelion, L. (2020, June 18). UK virus-tracing app switches to Apple-Google model. *BBC News*. Retrieved June 22, 2020, from https://www.bbc.com/news/technology-53095336.

[CR28] Lee, H. (2020, May 9). Tracing the problems with Singapore’s COVID-19 app. *East Asia Forum*. Retrieved June 8, 2020, from https://www.eastasiaforum.org/2020/05/09/tracing-the-problems-with-singapores-covid-19-app

[CR29] Lomas, N. (2020, 6 April). EU privacy experts push a decentralized approach to COVID-19 contacts tracing. *TechCrunch*. Retrieved June 18, 2020, from https://social.techcrunch.com/2020/04/06/eu-privacy-experts-push-a-decentralized-approach-to-covid-19-contacts-tracing/.

[CR30] Meaker, M., & Tokmetzis, D. (2020, June 23). Coronavirus apps show governments can no longer do without Apple or Google. *The Correspondent.*Retrieved June 23, 2020, from https://thecorrespondent.com/546/coronavirus-apps-show-governments-can-no-longer-do-without-apple-or-google/71545622148-0c7ce35d.

[CR31] Mol A (2008). The logic of care: Health and the problem of patient choice.

[CR32] O’Neill, P. H., Ryan-Mosley, T., & Johnson, B. (2020, 7 May). A flood of coronavirus apps are tracking us. Now it’s time to keep track of them. *MIT Technology Review. *Retrieved June 22, 2020, fromhttps://www.technologyreview.com/2020/05/07/1000961/launching-mittr-covid-tracing-tracker/.

[CR33] Pols J (2015). Towards an empirical ethics in care: Relations with technologies in health care. Medicine, Health Care, and Philosophy.

[CR34] Prainsack B, Buyx A (2017). Solidarity in biomedicine and beyond.

[CR35] RIVM. (2020, 8 May). Gedragswetenschappelijkeliteratuurrondmondkapjesgebruik. Een rapid review van de literatuur. Retrieved August 24, 2020, fromhttps://www.rivm.nl/documenten/gedragswetenschappelijke-literatuur-over-mondkapjes.

[CR36] Servick, K. (2020, 21 May). COVID-19 contact tracing apps are coming to a phone near you. How will we know whether they work? *Science*. Retrieved June 2, 2020, from https://www.sciencemag.org/news/2020/05/countries-around-world-are-rolling-out-contact-tracing-apps-contain-coronavirus-how.

[CR37] Sharma, P. (2020, 15 May). How has Mark Rutte performed during this crisis? *Holland Times*. Retrieved June 25, 2020, from https://www.hollandtimes.nl/articles/national/how-has-mark-rutte-performed-during-this-crisis/.

[CR38] Sharon T (2016). The Googlization of health research: From disruptive innovation to disruptive ethics. Personalized Medicine.

[CR39] Sharon, T. (2020, April 15). When Google and Apple get privacy right, is there still something wrong? *Medium.* Retrieved April 15, 2020, from https://medium.com/@TamarSharon/when-google-and-apple-get-privacy-right-is-there-still-something-wrong-a7be4166c295.

[CR40] Sharwood, S. (2020, May 4). Singapore to require smartphone check-ins at all businesses and will log visitors’ national identity numbers*. The Register.* Retrieved August 5, 2020, from https://www.theregister.com/2020/05/04/safe_entry_singapore_visitor_logging/.

[CR41] Terpstra A, Schouten AP, de Rooij A, Leenes RE (2019). Improving privacy choice through design: How designing for reflection could support privacy self-management. First Monday.

[CR42] Tokmetzis, D., & Meaker, M. (2020, 1 June). We were told technology would end Covid-19 lockdowns, but the truth is there’s no app for that. *The Correspondent.*Retrieved June 12, 2020, from https://thecorrespondent.com/502/we-were-told-technology-would-end-covid-19-lockdowns-but-the-truth-is-theres-no-app-for-that/66389901600-2c9929bb.

[CR14] van Dongen, A. (2020, August 14). Corona-app kanjuist tot meerbesmettingenleiden, zegtdeze Harvard-onderzoeker. *Het Parool*. Retrieved August 24, 2020, from https://www.parool.nl/gs-ba297a1e.

[CR43] Verbeek, P.-P. (2020, 2 May). Corona-apps en de frontlinie van de ethiek. *De Ingenieur.* Retrieved June 18, 2020, from https://www.deingenieur.nl/artikel/corona-apps-en-de-frontlinie-van-de-ethiek.

[CR44] Wassens, R. (2020, 17 April). Groeiende kritiek op 'gehaaste' selectieprocedure corona-app. *NRC.*Retrieved August 23, 2020, fromhttps://www.nrc.nl/nieuws/2020/04/17/greoiende-kritiek-op-gehaaste-selectieprocedure-corona-app-a3997130.

[CR45] WHO [World Health Organization]. (2020). Contact tracing in the context of COVID-19. Interim Guidance, 10 May. Retrieved June 19, 2020, fromhttps://www.who.int/publications-detail-redirect/contact-tracing-in-the-context-of-covid-19

[CR46] Wokke, A. (2020, 7 May). Nederlandse corona-app zal gebruik maken van api van Google en Apple.*Tweakers*. Retrieved June 24, 2020, from https://tweakers.net/nieuws/166878/nederlandse-corona-app-zal-gebruik-maken-van-api-van-google-en-apple.html.

[CR47] Yasaka TM, Lehrich BM, Sahyouni R (2020). Peer-to-peer contact tracing: development of a privacy-preserving smartphone app. JMIR MHealth and UHealth.

